# American Dermatological Association

**Published:** 1881-11

**Authors:** 


### Society Reports.

ARTICLE IX.

AMERICAN DERMATOLOGICAL ASSOCIATION: FIFTH ANNUAL MEETING. Held at Newport, R. I., August 30 and 31, and Sept. 1, 1881. Official Report of the Proceedings by the Secretary, Dr. Arthur VAN HARLINGEN, of PHILADELPHIA.

### FIRST DAY.—MORNING SESSION.

### ADDRESS OF THE PRESIDENT, DR. JAMES NEVINS HYDE.

**Dermatology and its Relations to Periodical Literature.**

GENTLEMEN OF THE ASSOCIATION:

In selecting as a theme for the annual address, one which covers the broad field described in the words, Periodical Dermatological Literature, I feel that at the outset I should recognize and impose upon it a necessary limitation. The two successive addresses of the distinguished gentleman who preceded me in this place, have furnished us with a valuable history of the rise and foundation of American Dermatology, including, under that name, a sketch of the current and completed periodicals published in this country in the interest of this department of medical science. Of those two addresses it may be said, that each is the fitting supplement of the other, and their publication, I might add, has attracted, beyond the borders of membership in this association, an interest which cannot fail to have an important bearing upon future contributions to the same general field.

It is my purpose, at this time, to briefly inquire, what have we a right to expect of Periodical Dermatological Literature in its attempts to supply the needs of the profession at large, with respect to a diffusion of knowledge as to the methods of establishing an accurate diagnosis in cutaneous diseases and as to the means of their successful treatment. It is true that these questions are those whose answers must be familiar to all of you, and with respect to which I could not claim to be able to speak with any greater authority than yourselves. Still, it is on occasions like these when he who speaks in your name speaks with an added emphasis and a weightier than his own authority, that our utterances are received with the greater attention by the general profession of which we are all members.

Periodical Dermatological Literature exists in two tolerably distinct classes of publications. On the one hand, we find a list of special journals devoted chiefly to the consideration of diseases of the skin and kindred topics ; and, on the other, the great body of current medical issues, conducted with a view to the advancement of general medicine or to its special departments distinct from that in which we find a special interest. The list of the former is limited and relatively small, not more than five or six of such journals now appearing in all countries, and of these none in Great Britain. The number of regular subscribers to these special publications is also necessarily limited ; and it might be safely concluded that their pages are consulted by a still smaller number of general practitioners from the beginning to the end of the year.

When we turn, however, to the vast number of journals of general medicine published here and abroad, we find in them the field from which most medical men derive their knowledge, limited or the reverse, upon dermatological subjects. It is to the value of the articles which appear in their pages that the profession is indebted for its brightest successes in dermatology; it is to the deficient and erroneous teachings of the same sheets that we have to look for much of the ignorance and many of the vicious practices with which the consultant has reason to become familiar. I desire to ask your attention to this state of affairs for a few moments. And let me premise what I have to say with the remark, that large as has been the value of the contributions made by the members of this association since the day of its organization to special journals of dermatology, it may well be concluded that the work the same gentlemen have accomplished, in extending a knowledge of diseases of the skin by their communications to general medical literature, has been of equal value to the profession at large. I do not, however, fail to appreciate the fact, that the general diffusion of knowledge regarding any special department of science is apt to be proportioned to the fruits of the labor expended in its particular channels of research.

Contributions of the class we have now in view, those, namely, which from their wider circulation secure a more extensive reading, may properly be assigned to four categories. There are, first, the solid and praiseworthy observations of fact, or deductions of a trustworthy character from the observations of others, respecting the etiology, diagnosis, pathology and treatment of diseases of the skin. There are, second, reports of improperly, because imperfectly, observed facts and crude opinions derived from these imperfect observations. One reads these with a feeling of regret that the author had not brought to bear upon his theme a riper experience or a more extended collaboration of the results of investigation made by others. Next, we have the worthless papers of men totally ignorant of the meaning of the terms and facts which they have attempted to employ, and of the significance of the relation of these to each other, or to others beyond the scope of their recognition. Unfortunately, the syntax and logic of these three varieties of literature are not always proportioned to their intrinsic value. If they were, the educated man might, from his general knowledge alone, be able to distinguish between the wheat and the chaff. Sometimes such proportion does exists, often it does not ; and a certain degree of education in dermatology is thus needed in order to detect the counterfeit of the truth, when the latter is presented in an attractive form and under the cover of a name of authority.

A fourth class of papers is negatively a source of mischief, worked, however, chiefly to the authors themselves. These are written by men who should know well the subjects which they attempt to discuss, if we judge them from the standpoint of position and experience ; but either from an undue haste, originating in the pressure of many duties, or from a foolish desire to identify themselves speedily with the subject upon which they write, or even from other motives not of the highest character, they deluge the medical press with voluminous papers filled with verbose iterations of ideas made public before their day, often by others in much better form, which serve to encumber the world with a little more of the rubbish swept annually by a pitiless indifference into the limbo of the forgotten.

Very much, however, has been accomplished in educating the mass of professional men in America with respect to the interesting and fruitful topic which we are now considering. An examination of the medical periodicals published in this country during the last five years, and a comparison of these with those of an earlier date, will bear evidence to the fact that in their appreciation of the importance of cutaneous medicine, in the accuracy of diagnosis and in the discussion of questions connected with obscure cases, there has been an improvement which has been as creditable for the past as it is promising for the future.

Permit me, however, to suggest very briefly some of the particular topics upon which there is room for a more extended knowledge among the mass of the profession, and where such information will make the immense difference between the brilliant success or the dismal failure of the average practitioner of medicine.

Surely it rests upon us to do our part in excluding from the discussion of the important questions presented to the minds of our generation, the ghosts and spectres of those which have preceded, and which have scared so many of our kin into the devious thickets on either side of the path of safety.

In the matter of diagnosis, for example, how common it is to find the forms of papular eczema mistaken for prurigo, merely because there was not the discovery of a vesicle, that ancient *sine quâ non* of an eczematous disorder. Here, again, is a supposititious eczema, long and vainly treated because of the failure of the practitioner to distinguish the lesions of pediculi inhabiting the clothing of the patient. I was lately summoned by one of the shrewdest physicians of my acquaintance for consultation in a case supposed to be one of obstinate eczema of the forehead. doubtless so designated by my friend in consequence of the vesicular character of the disease, which proved to be an exceedingly severe form of zoster ophthalmicus, the scars of which the unfortunate patient will carry to his grave. Erythematous eczema of the face as it occurs in middle-aged adults is another variety of the same disease whose recognition becomes too often a stumbling-block to the medical man clever enough to discover and relieve every species of laceration of the cervix uteri. Patients thus affected frequently drift about among physicians, now treated for "erysipelas," and now for some

"humor of the blood," often attributing their malady to the most remote and impossible of causes. The case of an intelligent lady occurs to me in this connection, who, when consulting me for an affection of this character, referred all her troubles to the incursion of an insect several years before into the respiratory passages, and who would consent to no remedial measures which did not contemplate the removal of the unwelcome and now venerable visitor.

Turning to another of the common diseases of the skin, acne, one would be disposed to conclude, that the ease with which it can be usually recognized, its frequent exposure in public to the eye of the uneducated man, would serve as a basis for a very wide-spread familiarity with its symptoms and character. Such, however, is, as you all too well know, far from the case. Truly, if we were less ignorant of things spread daily before our eyes, we should have less difficulty in apprehending the hidden and abstruse. Possibly, it is exactly because acne occurs so frequently upon the face, that so many odd errors are committed against its personality. Thus we find a patient who has at one time been infected with lues, and who has perhaps exhibited a series of syphilodermata, with a few tertiary lesions of periosteum or bone, leading a life of dissolute habits for eight or ten years thereafter, and subsequently developing one of the forms of rosacea about the nose, forehead or cheeks. The arguments necessary to convince the average medical man, that this is not a symptom of syphilis and is absolütely unconnected with the infective process, are sometimes scarcely within our reach. Fact and reason are often equally powerless to dissuade these individuals from pursuing for years a specific treatment, which is as often disastrous to the personal appearance of the patient as to the reputation of the practitioner.

Then, again, there is the case of the man who has been exposed to venereal disease in his youth, without having been really infected, the acne of whose maturer years is persistently regarded by himself as the pitiless revenge which time inflicts for his vicious employment of it in the past. A striking example of this class of cases once came under my observation. A respectably appearing gentleman, fifty-five years of age, and affected with severe acne vulgaris lasting for fifteen years, came to the consultation accompanied by his wife, to whom he had been married for a quarter of a century. Just before the appearance of the rebellious acne, she had discovered her husband in improper relations with a woman of questionable character, and for fifteen long years the wife had reproached the husband for a disease which she supposed had been communicated to him, and the evidences of which she thought she saw in his face. The proof of these assertions upon her lips, corroborated as they were by many physicians, he had never questioned. A most careful examination revealed the true state of affairs, and it occurred to me, that the triumph of the husband as he found the stigma of his disease removed, seemed even less than the disappointment of the wife at finding herself deprived of her accustomed weapon of attack. Lastly, there is the man or woman, who is sexually an hypochondriac, and to whom the existence of a facial acne is a source of terror, as significant in his or her eyes, and in eyes, too, that should be better instructed, of poisonous or vicious matter in the blood, produced by disease of the generative organs. In the futile effort to dissipate or antagonize these "impurities," medicaments are swallowed for months at a time, with results which are often such as to transform pustules into large abscesses of the sebaceous follicles, and to produce a consequent facial deformity, which, if we followed the fashion of our ancestors in giving long names to symptoms, we should denominate the "facies sexualis medicamentosa."

How singularly the popular belief as to the generation of poisons in the blood, accords with the creeds of mediæval theology respecting the vileness of man! In the purview of the latter, the living body was a corrupt and evil thing ; self-neglect, a duty ; self-mutilation, a virtue ; and self-immolation in desert or cell, the crowning glory of a perfect life. If it be charged that this is the age of the deification of matter, we may well retort that that was the day of the apotheosis of dirt! To it, I believe, may be traced the prevalent doctrine of blood impurities, as the voudooism of the American black is found to have its source among the traditions of the negroes in the heart of Africa. What more pitiful than to listen to a healthy man, the father perhaps of a healthy child, his face flushed with the ruddy hue of the current that sustains his life, able to digest roast beef and to enjoy sunshine, showing merely a few pimples upon his face, and describing his body in terms that would be appropriate in characterizing a pile of ordure! In cutaneous medicine, it may not be true that, as Huxley said, "the people perish for want of knowledge," but it is certain that, for want of it, they endure a great deal of wretchedness and misery.

Turning to the somewhat rarer varieties of skin disease, we find that the common errors committed in their recognition are often as ludicrous as they are mortifying. You doubtless recall the case mentioned by one of our number, at the New York meeting, who had been consulted respecting a supposed case of leprosy, which was in fact one of melanotic sarcoma. The late president of the association recently informed me that his opinion had been asked with reference to a patient who had been assured by his physician that he was the subject of leprosy, and who was found to be suffering merely from an attack of tinea versicolor. It is a common experience in the last-named affection to encounter patients who have been treated for years for the relief of hepatic or other visceral disorder, and yet who are restored to their normal condition in the course of a few days by appropriate treatment as soon as they fall into the hands of a competent physician.

Lupus, again, is a disease of the skin, whose name has been employed to cover a multitude of mistakes. The various forms of carcinoma are so little differentiated, in the understanding of many practitioners, and it may be said also of many surgical authors, not only from each other, but from other lesions of less grave portent, that they are frequently confounded under the designation lupus. The fact that lupus vulgaris is, at its outset, most commonly a disease of early life, is not generally appreciated beyond the dermatological world, and the result is that it has served as a convenient halting-ground for many who are called on to give an opinion respecting suspicious solitary ulcers of advanced years, the real nature of which is not even suspected. Thus we find it used very largely by the men who do not hesitate to align themselves with sectarians in medicine, and whose knowledge of half truths is apt to be proportioned to the minimum of their doses. It is none the less remarkable of lupus erythematosus, that it is not rarely confounded with squamous eczema, and when persistent, combatted by remedies supposed to be efficacious in the so-called diseases of the blood, such as sarsaparilla and stillingia, those innocent weapons of warfare, which ought one day to be displayed in an international museum of medical antiquities, to be gaped upon, wide-mouthed, by coming generations, as does our own upon the relics in the Tower of London.

I refer him who is interested in errors of etiology to the admirable paper on that subject, written by the first president of this association, and now a part of its record. It should be read by every American student of dermatology. Perhaps it may not be inconsistent with the theme I have chosen, to touch very briefly, at this point, upon the demands, implied or expressed, upon the exponents of cutaneous medicine, with respect to the important relation they sustain to periodical literature.

It is well-nigh impossible to mistake the drift of the wisest popular and professional thought, in its influence upon the practice of our time. I believe that, erroneous as are many of the conclusions in the philosophy of Mr. Herbert Spencer, they have powerfully operated to inspire the modern methods of research with a keener appreciation of the real relations sustained by man to the external universe, and the influence of those relations upon each of his physiological and morbid states. Studied in its proper light, the altruism by which Mr. Spencer would explain man's moral sense, and even his affection for his offspring, becomes the key to many of his diseases and to the most rational methods of their control. Man is thus discovered, with the devils by which he was formerly possessed cast forth, and he stands the innocent or guilty victim of the folly, ignorance or vice of himself or another. His sufferings, no less than his passions and emotions, become links in a long chain of causes and effects. Absolution for his physical sins of omission and commission can not be purchased by swallowing a pill or annointing himself with a salve, as readily as the burdens of his conscience are purged by the pardons of a priest. The attractiveness which the study of hygiene possesses for the modern investigator lies in its revelation of the possibilities of preventive medicine, if you will permit the use of such a misnomer, and I do not know of any field which, in this regard, is so promising as dermatology.

It is beginning to dawn upon the minds of men that it is easier to hurt than to heal; cheaper to foresee than to suffer; wiser to be prevented than to be punished.

We have sadly perverted, in these latter days, the primitive sense of the old Latin word *curare*—to take care of, or to care for ourselves or others. We have made it mean that part which man plays in the healing of the diseases of his fellow-man, a most detestable and wretched rendering of the original, with which the Italians are not chargeable. They still use the word *cura* in the sense of the care or treatment of a malady, while the verb *guarare* is applied to the healing or complete relief which is obtained by the cure. We find the term occasionally creeping into our own tongue with its honest meaning, as in the so-called grape-cure, or grape treatment of one class of maladies, and in like expressions. The true physician is really called on to cure, not only the sick, but also the well ; to care for both, in the highest sense of the term. by preventing all their preventable disorders, and, in those which are not preventable, by preventing all preventable complications.

As for our prevalent employment of the word cure, it is safer to look upon it with a pessimistic than an optimistic eye. It is not only certain that in this sense we cure nothing, it is even probable that no inflammatory disorder of severity is ever perfectly "cured," even by the elegant processes of nature. We are dying from the cradle to the grave. These so-called diseases of ours are in reality steps toward the final dissolution, arrested or not, but never retraced. Do you turn to me and say, "This is ground which a dermatologist should, of all men, hesitate to assume?" I answer that what can be shown to be the law in one department of knowledge, will probably fit well into all others. Does a cured pneumonitis leave the affected lung as distensible and pervious as before? How often will a cured pleuritis delicately glue together the apposed membranous surfaces in a union which only the hand of the anatomist will sever? The extremities of a fractured bone are united, it is true, but we can read the story of the accident in the skeleton for generations after. What blennorrhagia leaves the urethra of the male, virgin of all accidents? When we turn to the skin, and discover that the prick of the lancet which penetrates beneath the mucous layer, tearing asunder a few of the epithelia, leaves there the indelible trace of its entrance; when we observe that the most perfect coaptation of the lips of a wound, made by a clean incision of the skin proper, is followed by the formation of a new growth which no trick of man can remove, save by the substitution of another more cumbrous in its place; and when we reflect that scars, even half a century old, may reopen under the influence of scurvy, we may well ask of the presumptuous physician whether he will undertake to do better than all this?

We may look upon scars as the happiest results of natural effort to relieve a serious disease, and our best is scarcely better than that. What nature accomplishes with grace, delicacy and leisure, we effect brutally, coarsely and with despatch by knife, écraseur, curette or caustic. Let us call it "a cure," if we choose. It is really an arrested necrotic process, and the influences which decide whether that arrest shall be permanent or transitory are practically beyond our control.

This wonderful and complex organism which we call the skin is a living and sentient veil, stretched between the inner man and the outer world, with a series of complex organisms on either hand. However much we may conscientiously differ respecting the relative influence of the one side, or the other, in the production of its diseases, we can not fail to recognize the important fact of its interposition between the two. Even in those disorders which are admitted to be largely of internal origin, we discover that their study can not rest with the recognition of a single etiological factor, but must be pushed further to the investigation of those accidents which operate to give form, outline and position to all significant lesions. Thus, for example, Parrot has recently called attention to the fact that in hereditary syphilis the frequent occurrence of mucous patches about the anus is due to the accumulation over that part, of the fæces on the napkin of the child, and the labial sores of the same little patients are known to originate in the action of the lips on the nipple of mother or nurse. The close observer will be constantly surprising these external influences in their operation upon the living body, in every variety of pathological condition; and it would be as unphilosophical to assign the bed-sore of typhoid fever to the emanations from a cess-pool, as to conclude that the patches of tinea versicolor on the chest of the tuberculous patient were the result of a diathesis.

In this fragmentary way, I have merely alluded to a few of the special topics to which this subject naturally directs our attention. Will you permit me to formulate, with a view to their distinct expression in this place, a few of the conclusions which are thus suggested? Some of the common errors, then, which require refutation, are:

First. That because a disease exists and is visible on the surface on the body, it for that reason alone requires active internal or external treatment. Surely the diseases of the skin share the peculiarities of those of many other organs of the body. Some need no treatment. He is, indeed, well fitted for his responsible position who advises his patient that the disease of the latter disturbs the imagination more than the body; is possessed of no element of danger to himself or another; will, if undisturbed, proceed naturally to its own favorable issue, and call for no intervention on the part of either physician or patient. These cases occur to all of us in our daily experience. I had, lately, the fortune to watch the evolution of forty cases of rötheln, medically untreated in one of the public charities of Chicago. In no one of these was any drug administered, and in none did any accident occur worthy of mention, beyond the usual features of the disease.

Second. That because a disorder of the skin exists, it can be always readily relieved by an expert. There are cutaneous maladies which prove inevitably fatal. There are others of such an inveterate character that the skill of the most practised is inadequate to procure relief. Though not fatal, the latter are none the less persistent and remediless. Is it not curious that there should even be occasion to remind the world that the wisdom and skill of man have not yet reached the acme of success in all those emergencies where a demand is made upon the resources of the healing art? Are there no forms of non-specific catarrh which resist the most conscientious efforts of the physician? Are there yet no deformities which the skill of the orthopædic surgeon fails to remedy? There are thousands of men to-day on the pension rolls of the United States Government for chronic diarrhœa, which has been uncontrolled since its victims first contracted the disease in the swamps of the Southern marshes. It may well be regarded as an unintended compliment to the skill of the dermatologist that he is ever and again consulted for the relief of a cutaneous disorder which has lasted for sixteen or twenty years, and which he is asked to remedy in the course of as many days. "A chronic disease," said Fournier, "requires a chronic remedy;" but, in the view of the uninformed, this axiom is reversed. There are inveterate diseases of the skin, as of other organs of the body, and, brilliant though be the success with which some of these are managed by the competent man, a variable proportion will bid defiance to the most practised hand, guided by the best trained judgment, till our methods are improved and our resources largely increased.

Third. That after relief is secured by treatment, many diseases of the skin can be so managed that they will not recur. The implied demand is made, it seems to me, upon the dermatologist alone. The patient with a fractured leg does not ask of his surgeon a guarantee that he will not suffer a similar accident, if he again fall beneath the wheels of a rolling car. The convalescent from a follicular enteritis, does not demand a future immunity from errors of diet in an unfavorable season or unpropitious climate. And so the patient, with an intertrigo beneath her pendulous breast, or a dermatitis medicamentosa, should not expect to be protected from the results of either a neglect or an imprudence. Some diseases of the skin recur continually, and will do this for a lifetime. It is true that these recurrences usually result from adverse extrinsic influences, but it is often not in the power of the physician to set these aside. In such cases he discharges his full duty, when he forecasts the future and describes the result of the best treatment under forbidding circumstances.

Fourth. That the expert, upon the examination of but a portion of the affected integument, is competent to pronounce at once upon the character of a disease. It is true that in many cases a large experience enables such a man to recognize at a glance the distinctive features of a malady. In the same way the surgeon can occasionally, and with equal readiness, recognize a case of talipes, and the oculist one of convergent squint. But no opinion is worth the time of the patient or consultant, which is not based upon a most careful, exhaustive and patient investigation, not only of the visible symptoms present to the eye upon the surface, but also of the history of the case and all other pathological facts obtainable. It is, indeed, the skill with which these are discovered and assigned their proper influence in a decision as to the nature of the complaint, that constitutes the chief difference between him who is, and him who is not, expert in diagnosis. Strange, indeed, that the dermatologist alone should be supposed absolved from this imperative and fruitful labor in the study of his cases, yet such an idea is sufficiently wide-spread, largely among the laity, and to a certain extent among the profession. It is a common experience to be shown a patch of diseased skin or a few distorted hairs, upon which follows the inevitable question, "What do you call this?" I was lately stopped on the street by a physician of large practice and wide reputation, doubtless on the way to the bed-side of his patient, who hurriedly demanded, "Please tell me how to treat eczema!"

Fifth. That a practicing physician need not always make an accurate diagnosis in cutaneous disease, and that a failure on his part to do this, need not be considered as perilous to himself. In a recent inaugural address, delivered before the class of one of the medical colleges in the city of New York, * the speaker remarked, "The physician must never forget that his patient is, from the nature of things, a possible enemy." Well and truly said. The time has nearly passed when our intelligent countrymen, seeking to know the nature of their complaints, can be put off with a long-sounding name and a prescription written in a dead language. For many of them it will no longer suffice to be informed, in the matter of diagnosis, that they are affected with "a disease of the skin." They ask, many of them, to know what form of skin disease is theirs, and, if not satisfactorily answered, it requires but little to effect this transformation of a friend into an enemy. The fact will finally dawn upon them that they have been in charge of one who really could not even name the disease which he had dared to treat. "What," they properly exclaim, "would be thought of a surgeon who would undertake to treat a fracture, not knowing whether it were a dislocation or a rheumatic affection of a joint, and what would be the position of a merchant who, in the commercial world, could not exactly name the amount of his liabilities?" Sooner or later, patients of this class find out for themselves the persons who are competent to describe their condition and to advise them judiciously with

---

* Inaugural Address. M. Putnam Jacobi, M.D., CHI. MED. JOUR. AND EXAM., June, 1881, p. 561.

respect to its management. We, who often are consulted at this juncture, are sometimes compelled to listen with a feeling of shame for our profession, to indignant reproaches on the lips of those who, but a brief time before, trusted to the honor and relied on the skill of our brothers in medicine. It is none the less pleasant to record the attested fact, that the man who is candid enough to admit that he is in doubt respecting the nature of the disease for which he is consulted, and who summons another, in order to avail himself of a wider experience or a readier skill, rarely fails by such a course to strengthen those bonds of sympathy which should always unite the physician and patient. In the vast majority of these cases, patients return to their physicians with an heightened sense of the trustworthiness of the latter.

Let no man interpret these statements in the light of a plea for a wider range of possibilities for the consultant. The educated gentlemen who have made themselves masters of any special department of science, in this country, certainly have found no lack of appreciation and that reward for skilled labor which all honest work inevitably yields. They surely need to have no plea entered for them. This association has steadily set its face in the direction of the needs of the physician in general practice with respect to a knowledge of cutaneous diseases, and has made a most important and valuable advance in the diffusion of such knowledge in America. If these words of mine can be interpreted as a plea, may they be held as pleading for higher attainments on the part of the general practitioner.

Sixth. That such remedies as mercury, arsenic and the iodide of potassium may be administered in any case where an accurate diagnosis has not been established, and in such cases may be used unadvisedly. Said the physician of a past generation, "When you do not know what else to do, treat for syphilis." It is difficult for me to touch upon this point, without the appearance of exaggeration. Doubtless the experience of many of you does not greatly differ from my own. I am quite prepared to say, that for one patient who is benefitted by the administration of these remedies in the hands of incompetent men, there are ten who are made worse. The vulgar idea that all disorders of the integument are, in some obscure way, to be connected with a syphilitic taint in one generation or another; the erroneous opinion that syphilis descends through many generations and lurks obscurely in the eczema of the infant, the psoriasis of the adult, the sycosis of the young man, or the acne of the young woman, these are the spectres that scare the foolish, whether they call themselves doctors or laymen, into the rough régime of a crude materia medica. It should be considered a crime to subject a man or a woman to a systematic mercurial course for the relief of an affection supposed to be syphilitic, but in reality not such. The aggravations induced in acute cutaneous exudations by the early and indiscriminate employment of arsenic, need not be described to those who, like yourselves, have the opportunity of observing these conditions. Acne-sprinkled faces, which for months have exhibited almost every one of the various eruptions induced by the ingestion of the iodide of potassium, are the staple of our public clinics and the reproach of our body medical. Who will write, in figures that should astonish the student of medical statistics, the number of all diseases of the skin, readily manageable at their outset, but after long subjection to the so-called "driving-out" process, rendered either inveterate or remediless?

Seventh, and lastly. That the study of diseases of the skin is the pursuit of a narrow specialty, leading to a knowledge of superficial eruptions upon the surface of the body, and to an ignorance of the functions and maladies of important internal organs. The erroneous impression thus suggested has contributed to the too general disregard of cutaneous medicine, not only in the medical colleges and charities of our country, but also, in part as a consequence of this, in the studies of professional men. Yet he who would specially devote himself to the investigation of the disorders of the skin requires, fully as much as his professional associates, the amplest preparation in a finished general medical education and experience; and when at last ready for his work he will discover, if he has not before done so, that he needs to be a physician in the highest sense of the term, and the peer of all his fellows. There is no organ of the body, either in health or disease, which it may not become his duty to explore and investigate, for the skin is only one of the living and potential microcosms which constitute the macrocosm of the human frame. Surely I need not remind any of you, that he can ill afford to neglect the study of diseases of the skin, who devotes himself especially to the maladies of the eye, of the ear, of the generative organs of either sex, of the nervous or other system of the living body. The same is true of the surgeon, the accoucheur, and of the professional man who is called to treat the disorders of infancy and childhood.

The added power and skill of him who acquires the knowledge of which I speak is something which its possessor alone can fully appreciate, and of him who has it not, I think you will agree with me in saying, that as a diagnostician he is fatally deficient. Who can describe to one that knows nothing of it, the sense of gratification afforded by the ability to read at will the page presented on the surface of the living body, its secrets utterly sealed to another, who finds these characters meaningless! Who will declare the marvellous manner with which nature rewards such a man for his patient, minute and watchful attempts to translate these characters, giving them a clearer significance and more legible distinctness for every added hour of attention! Who, that has once enjoyed this enlarging reward for his toil, would exchange it for the power to decipher any hieroglyphics that were ever graven by man upon a monolith, or imprinted by his pen upon a palimpsest! His is a truer palmistry than that which seeks only to read the secrets of the inner man; for to him the human hand, since first the pink of its unused palm was laid upon the breast of a mother—and whether it betray the syphilis a decade old, or the manner of toil by which man earns his bread—tells ever a true tale of its acquaintance with that part of the world which it was fashioned to grasp.

In the hope, Gentlemen of the Association, of making this address of some practical value, and also of further justifying its title, I have appended to it a general alphabetical index, by authors and subjects, of all the original communications which have appeared in the pages of our special dermatological journals, including also the papers written by members of the association and published elsewhere. The index, therefore, is made from the following periodicals :—

The *Vierteljahresschrift für Dermatologie und Syphilis*, Wien, 1875 to July, 1881. Current.

The *Archives of Dermatology*, New York, 1874 to July, 1881. Current.

The *Archiv für Dermatologie und Syphilis*, Wien, 1869–1873. Complete.

The *American Journal of Syphilography and Dermatology*, New York, 1870–1874. Complete.

The *Annales des Maladies de la Peau et de la Syphilis*, Paris, 1844–51. Complete.

The *Annales de Dermatologie et de Syphiligraphie*, Paris, 1869 to July, 1881. Current.

The *Archiv für Syphilis und Hautkrankheiten*, Berlin, 1846. Complete.

The *Giornale Italiano Delle Malattie Veneree E Delle Pelle*, from the 11th year to July, 1881 (the first ten volumes being omitted.)

The *Journal of Cutaneous Medicine and Diseases of the Skin*, London, 1868–1871.

The index, thus prepared, is, I am aware, too large to be incorporated with the proceedings of this association. I shall therefore hope at no distant day to place it in the hands of the members of the association in a printed form, without adding to the volume or the expense of our annual transactions.

The President's address having been delivered, DR. CHARLES HEITZMANN, of New York, read a paper entitled

### A Contribution to the Minute Anatomy of the Skin, of which the following is an abstract:

The apparently complicated structure of the skin is easily understood if it is kept in mind that there are but two main tissues entering into the structure, viz., connective tissue and epithelium. The former constitutes the derma, and is arranged below in a coarse felted network and above in fine delicate fibers without distinct arrangement. The superjacent epithelial layer is subdivided into two strata, the lower (rete mucosum) alive and supplied with nerves, the upper (epidermis) of dry, horny epithelia. The connective issue is supplied with blood-vessels and lymphatics, the epithelium lacks such structures.

If, now, considering the formation of the hair, we imagine the connective tissue and epithelium together as a pliable sheet as of chamois, and if in this a depression is produced by pressure with the end of a finger, the result will be a pouch, the innermost layers of which are epithelial, while the outer layer is connective tissue. The epidermis covering the inner surface of the pouch now bears the name "inner root sheath." Next to this will be a layer formed by the epithelia of the rete mucosum, the "outer root sheath." The outer portion of the pouch composed of connective tissue represents the "follicle." On the bottom of the pouch a protrusion upwards entirely analogous to the papillæ, and formed like those of connective tissue, constitutes the papilla of the hair.

In the formation of the pouch the horny epidermic layer is much thinned in its involution, becoming attenuated in the middle to two rows of cells, thickening again lower down to three or four strata of epithelia, which have lost their horny character and have again become protoplasmic. The rete mucosum enters the pouch in full width, but becomes narrower ; the cells, however, retaining their protoplasmic character, until it is reduced to a single layer and finally disappears.

If we imagine the bottom of the pouch pushed upward, this prolongation, from the structure above described must consist of the inner-root sheath : it represents the hair, which is therefore a solid elongation of the hollow inner-root sheath ; the outer root-sheath has nothing to do with the formation of the hair. At the bottom is the bulb of the hair, composed of living protoplasmic epithelia. Higher up in the hair the epithelium becomes horny again to form the shaft of the hair.

The writer went on to describe the formation of the sebaceous follicle from the outer root-sheath ; the inner root-sheath has nothing to do with this formation. The pouch of the sebaceous gland is under the control of the arrector pili muscle, which assumes the form of a flat fan-like sheet, the broad ends of which terminate in the papillary layer, while the narrow end is inserted into the follicle. No doubt the evacuation of the sebum is effected by the contractions of this muscle, the fatty matter being squeezed into the funnel of the hair pouch and not directly out to the surface excepting in the largest glands.

After giving some further account of the minute structure of the hair and of the process by which it is shed and renewed, the writer went on to describe the formation of the sudoriparous glands. We may simply assume a prolongation of the outer epithelial layers into the deeper portion of the derma. In the epithelial layer of the skin, where the cork-screw-like windings are observed if this is thick, as on the soles, the sweat duct is lined with flat epithelia. At the point where the duct pierces the rete mucosum, in the valley between two papillæ, it is lined by stratified epithelium. Within the derma, the duct is composed of a single layer of columnar epithelium, obviously a prolongation of the lowest layer of the rete mucosum. The tube, as it begins to coil up, is lined by a single layer of cuboidal or short columnar epithelium. Both the duct and the coil are invested by a somewhat denser connective tissue and supplied with blood vessels. Between the connective tissue and the epithelium there is sometimes a distinct structureless or hyaline layer. Larger coils, especially those of the axilla are supplied with smooth muscles, which are imbedded in the ensheathing connective tissue capsule.

DR. LOUIS A. DUHRING, of Philadelphia, congratulated Dr. Heitzmann on his paper, remarking that the facts there presented would greatly simplify the heretofore complicated views as to the relations of the various structures of the hair and its follicle.

DR. JAMES C. WHITE, of Boston, asked Dr. Heitzmann how it happened that he had never before observed the arrangement of the various layers as described in his paper.

DR. HEITZMANN said that he, like others, had formerly followed the recognized authorities. Recently he had gone to work independently. In answer to a question put by DR. EDWARD WIGGLESWORTH, of Boston, as to the formation of the hair pigment, Dr. Heitzmann further remarked that he had not as yet entered into the study of the subject.

DR. JAMES C. WHITE, of Boston, read a paper on

**The Limitations of Internal Therapy in Skin Diseases.**

The writer said he would like to approach the consideration of the subject from a purely empirical stand-point, uninfluenced by theories and traditions, and supported only by the results of observations made and recorded in ways recognized as strictly scientific. Cutaneous pathology offers an exceptional field for the study of drugs introduced through the stomach; for, in addition to the ordinary means of judging their effect, the phenomena of tissue-change or modification of function occur directly under the eye. Yet cutaneous internal therapeutics is no more fixed or satisfactory in its results than the therapeutics of general medicine. In spite of great activity and the fact that novelties even called specifics in skin diseases are scattered all along the history of medicine, only a few agents have attained and preserve a well-established reputation as serviceable in limited fields of usefulness. Dermatologists of repute in different countries still adhere to certain remedies from motives of conservatism or patriotism, while those of other countries cling to their pet drugs and regard foreign treatment as obsolete and erroneous. This unsatisfactory and contradictory condition of affairs must continue until better methods of study are adopted and the faults of individualism eradicated.

It would not be boastful to say that nowhere does there exist a body of dermatologists so broadly cosmopolitan in spirit and training, and representing such varied opportunities of research as are afforded by wide differences in climate and race, or one more competent for such work, than the gentlemen who compose the American Dermatological Association.

The writer went on to sketch an imaginary committee who should conduct a systematic investigation of new drugs. When a member of the association should present a paper on the specific effect of some drug in this or that affection, this would be submitted to the committee, who should follow some well-established procedure in testing its merits. In this way the association would do much to discourage the loose writing and the claims based on insufficient observation which generally herald the introduction of each new remedy. To such a committee might be referred not only all appropriate communications presented to the association, but all doubtful questions of importance in therapeutics, which now vex us so frequently, of old or new origin.

The following subjects of investigation might be cited by way of illustration:

1. The action, beneficial or injurious, of our most celebrated springs in affections of the skin. A report on this subject would do much to clear away the atmosphere of charlatanism which has hitherto obscured it, and develop some knowledge of certain characters regarding it, which the profession of this country has so long needed.

2. The power of electricity over the so-called neuroses of the skin (including zoster).

3. To what extent may certain substances in common use be applied to the skin without danger, viz., compounds of zinc, lead, mercury, carbolic acid, etc.?

It is unfortunate that so few beds in hospitals are under our control, and that we must rely upon the voluntary visits of out-patients. But, even with such opportunities only at our command, much may be done.

The speaker then went on to speak of the vague and meagre knowledge possessed regarding the action of drugs on diseases of the skin, as illustrated in systematic treatises, going over each class of diseases *seriatim*, and stating the amount of exact knowledge possessed as to the influence of drugs internally administered on each. The number of individual affections of the skin recognized by us approaches closely one hundred. How many of these are under the direct control of internal medication in any marked degree? The speaker wished he might claim such power over ten of them—such power as arsenic may exhibit in in psoriasis, as mercury almost always does in syphilis, and which makes external treatment unnecessary.

The writer did not mean to deny that much may often be done to hasten their good work by the simultaneous use of remedies which elevate the general tone of the patient's functions and tissues, when needed, together with local treatment. The whole patient must always be considered. Moreover, the writer did not believe that the narrow limit of our exact knowledge on this subject could not be enlarged. He would encourage any experimental studies, empirical or philosophical, in this direction. It is the manner in which this important subject has been hitherto so generally treated, so detrimental to scientific progress, which he deprecated. But a frank confession of our present inabilities is an essential step in any real advance.

DR. HEITZMANN, of New York, expressed his pleasure at hearing such views expressed. Men like Erasmus Wilson had done serious injury to dermatology by hastily asserting the influence of internal medication in diseases of a purely local character and indiscriminately.

### FIRST DAY.—EVENING SESSION.

DR. ARTHUR VAN HARLINGEN, of Philadelphia, read a paper on

### A Case of Lymphangioma Tuberosum Cutis Multiplex, giving an account of a woman, 30 years of age, who displayed a large number of tumors of various sizes and shapes distributed here and there over the whole body, together with numerous teleangiectases and irregular brownish patches of pigmented skin. The tumors in some cases resembled flabby molluscum fibrosum growths, while in other cases they were smooth lilac or bluish elevations from pin-head to hazel-nut size, and so compressible under the finger as to feel like bladders filled with air and to give the sensation of an umbilical hernia in a child. On excision they were found to consist of a pearly gelatin-like semi-transparent mass, with a smooth, bulging surface and without fluid contents. Microscopic examination showed the structure to be composed of fibrous and granulation-cell tissue, with numerous irregular spaces,—sections of dilated lymphatic vessels. But two other cases are on record, one described by Kaposi in Hebra's work on Skin Diseases, the other reported by Pospelow in the *Vierteljahresschrift für Dermatologie und Syphilis.*

DR. HEITZMANN, of New York, said that the writer was mistaken in thinking that only two cases had been reported. Billroth had reported a case of large lymphangioma, and a student of his had collected and published a number of similar cases.

DR. VAN HARLINGEN replied that he had examined the article alluded to and considered the cases of a different character from that he had just described.

THE PRESIDENT, DR. HYDE, of Chicago, alluded to Dr. Busey's work on the subject of disorders of the lymphatics, remarking that in the cases there reported, an escape of lymph, sometimes to a debilitating degree, was reported to have taken place.

DR. I. E. ATKINSON of Baltimore, asked the writer how he distinguished the molluscoid tumors described from true molluscum fibrosum, and went on to speak of cases where the tumors of true molluscum fibrosum could be pushed down into the skin as in Dr. Van Harlingen's case.

DR. WIGGLESWORTH reminded the writer of a case of molluscum fibrosum described by himself several years ago, in which several symptoms were observed of a similar character to those noted in the paper just read, notably the compressibility and the gelatinous appearance on section described by Dr. Van Harlingen.

DR. LOUIS A. DUHRING, of Philadelphia, read a paper on

**The Small Pustular Scrofuloderm,**

consisting of a report of three cases of the disease, together with a general description of the symptoms of the process, the probable cause, and the diagnosis. It may be briefly described as being characterized by pinhead and small split-pea sized yellowish pustules with a more or less raised violaceous fixed base. The lesions are disseminated, and usually appear on the extremities, especially the hands and forearms. In general appearance they resemble those of the small pustulæ syphiloderm. They pursue a slow course, and in the course of several weeks begin to crust over with a small hard or horny, grayish or yellowish, adherent crust, which in time drops off, leaving a marked, punched-out looking scar. The lesions appear at irregular periods, new ones coming out from time to time, the whole process lasting months or years. Symptoms of general scrofulosis are usually present, and hence the disease was supposed by the writer to be a cutaneous manifestation of scrofula. It is liable to be confounded with syphilis, with acne, and with follicular lupus. The follicles, however, here are not invaded primarily. The writer stated that the disease had been first described by him in the second edition of his Treatise on Diseases of the Skin, and that it presented such marked features and was of such importance as to be worthy of careful study.

Dr. HEITZMANN, of New York, asked Dr. Duhring if he was familiar with the clinical appearances of the acne cachecticorum of Hebra. So far as he could judge from the account of the cases just given, there seemed a close resemblance between the lesions there described and those of acne cacheticorum.

DR. DUHRING replied that he was familiar with the acne cachecticorum of Hebra, both clinically in Hebra's wards and by study and examination of pictures of the disease. The eruption was quite different from that which he had just described. In reply to a question by Dr. White, Dr. Duhring said that the follicles were not involved in his cases.

DR. J. C. WHITE, of Boston, said, speaking of the etiology of the affection, that he doubted if scrofula could influence the skin to such an extent.

DR. GRAHAM, of Toronto, mentioned a case similar to those reported by Dr. Duhring, which had come under his observation.

[CONCLUDED IN DECEMBER NUMBER.]

